# Poultry Concentrated Animal-Feeding Operations on the Eastern Shore, Virginia, and Geospatial Associations with Adverse Birth Outcomes

**DOI:** 10.3390/healthcare10102016

**Published:** 2022-10-12

**Authors:** Antonia Mendrinos, Balaji Ramesh, Corrine W. Ruktanonchai, Julia M. Gohlke

**Affiliations:** 1 Department of Biological Sciences, Virginia Polytechnic Institute and State University, Blacksburg, VA 24061, USA; 2 Department of Population Health Sciences, Virginia Polytechnic Institute and State University, Blacksburg, VA 24060, USA

**Keywords:** concentrated animal-feeding operations, preterm birth, low birth weight, spatial analysis, birth outcomes, air pollution

## Abstract

Concentrated animal-feeding operations (CAFOs) emit pollution into surrounding areas, and previous research has found associations with poor health outcomes. The objective of this study was to investigate if home proximity to poultry CAFOs during pregnancy is associated with adverse birth outcomes, including preterm birth (PTB) and low birth weight (LBW). This study includes births occurring on the Eastern Shore, Virginia, from 2002 to 2015 (N = 5768). A buffer model considering CAFOs within 1 km, 2 km, and 5 km of the maternal residence and an inverse distance weighted (IDW) approach were used to estimate proximity to CAFOs. Associations between proximity to poultry CAFOs and adverse birth outcomes were determined by using regression models, adjusting for available covariates. We found a −52.8 g (−95.8, −9.8) change in birthweight and a −1.51 (−2.78, −0.25) change in gestational days for the highest tertile of inverse distance to CAFOs. Infants born with a maternal residence with at least one CAFO within a 5 km buffer weighed −47 g (−94.1, −1.7) less than infants with no CAFOs within a 5 km buffer of the maternal address. More specific measures of exposure pathways via air and water should be used in future studies to refine mediators of the association found in the present study.

## 1. Introduction

Concentrated animal-feeding operations (CAFOs) emit ammonia, hydrogen sulfide, odors, volatile organic compounds, and particulate matter into adjacent environments [1]. Populations exposed to these chemicals may be at risk for a host of adverse health outcomes. Exposure to airborne ammonia and volatile organic compounds can aggravate lung function and may cause chronic lung disease [2,3], while hydrogen sulfide can cause inflammation of the eyes, nose, and throat [4]. Particulate matter may worsen lung function and can cause irregular heartbeat, heart attacks, and other cardiac abnormalities [5,6]. Lastly, changes in mucosal immune function, specifically in immunoglobin A responses, have been associated with odor intensity in individuals living in close proximity to farming operations, suggesting an immunosuppressive effect [7].

According to the Environmental Protection Agency, there has been a 16% increase in CAFOs in the United States from 2011 to 2020 (18,540 CAFOs to 21,465 CAFOs) [8]. Virginia’s Eastern Shore, consisting of Accomack and Northampton counties, has numerous poultry CAFOs, which have similarly increased in number and size from 254 chicken houses on 51 farms in 2014 to 480 chicken houses on 83 farms in 2019 [9]. In 2020, 70 farms in Accomack County had the capacity to produce 85 million birds per year and produce about 137,000 tons of manure [9]. Furthermore, a study commissioned by the Chesapeake Bay Foundation reported that 33.8 million pounds of ammonia is released each year from more than 600 poultry houses on Maryland’s Eastern Shore within the Chesapeake watershed [9]. From the 33.8 million pounds of ammonia, 22.5 million pounds was deposited back into the Eastern Shore’s terrestrial and aquatic ecosystems [9]. Despite the increasing prevalence of poultry CAFOs on the Eastern Shore and the evidence of their contribution to air and water pollution, few studies have assessed the health outcomes of nearby residents, particularly among vulnerable populations such as pregnant women and newborns. 

The etiology and pathophysiology of preterm birth (delivery <37 weeks [10]) and low birth weight (birth weight <2500 g [11]) is not well understood; however, evidence suggests that exposure to air pollution contributes to preterm-birth and low-birth-weight outcomes [12,13,14,15,16]. Maternal exposure to particulate matter has been associated with placental impairments such as inflammation, hyper-coagulability with vascular thrombosis, and increased production of free radicals and reactive oxygen species [17]. Maternal exposure to fine particulate matter has also been shown to increase the expression of IL-4, causing placental inflammation, which may impair the gas and nutrient exchange [18]. A reduction in fetal skull size and growth has been associated with maternal exposure to particulate matter, suggesting that exposure to air pollution may underlie low birth weight [19,20]. In addition, more than 3% of all premature births in the United States have been attributed to air-pollution exposure, impacting approximately 16,000 babies per year [21]. Complications of preterm birth may include lung, heart, brain, and immune impairment [22,23,24], and such complications have also been linked to diminished cognition and memory and poor white-matter organization [25,26]. Similarly, low birth weight may increase the risk of psychological problems, cognitive deficits, and neuromotor functioning [27,28]. 

Previous epidemiological studies in North Carolina and Wisconsin have found associations between living in close proximity to hog and poultry CAFOs and newborn mortality and low birth weight (LBW) [29,30]. Furthermore, a 2020 study exploring the geospatial association between hog and poultry CAFOs and birth outcomes in North Carolina found that mothers living within 2–5 miles of a poultry CAFO had 1.13 greater odds of preterm birth and 1.14 greater odds of LBW infants compared to those living 5 miles or greater from a CAFO [31]. Despite this, studies assessing the dose–response relationship between adverse birth outcomes and increasing proximity to poultry CAFOs generalized to other geographic regions are limited. With the increasing number and size of various CAFO operations across many rural communities in the United States, including Virginia’s Eastern Shore, the knowledge gap of how these operations impact local health is particularly relevant. 

Here, we aim to build upon the existing literature by exploring the potential maternal health implications of living near poultry animal-feeding operations on the Eastern Shore, Virginia. Specifically, we use the address of maternal residence on state birth records, capturing births in Accomack and Northampton counties, from 2002 to 2015, combined with the locations of active poultry farms during each year of the study period. We hypothesized that birthweight and gestational weeks were negatively associated with increasing proximity to active poultry CAFOs during gestation, with increasing odds of preterm birth and low birth weight. 

## 2. Materials and Methods

### 2.1. Birth Outcome Data 

This study utilized birth records occurring within Virginia’s Eastern Shore (Accomack County and Northampton County), from 2002 to 2015, provided by the Virginia Department of Health. Each record consisted of maternal residential address; birth plurality; parity; newborn weight and gestation; self-reported tobacco use during pregnancy; type of payment used for birth services; and maternal race and ethnicity, age, and education level. Singleton births with no unknown or missing covariates were utilized in the analysis. Missingness accounted for less than 1% of records (Table 1 and Supplementary Table S1). Maternal residential addresses recorded at birth were geocoded to the street level by using ArcGIS software [32], using the open-source 2013 OpenStreetMap file [33], resulting in a total of N = 5768 birth records. A separate analysis was performed at the ZIP-code level (N = 7306 birth records) to account for the significant number of birth records without an identifiable street address (such as P.O. Box addresses, N = 1538 birth records). P.O. boxes accounted for 21% of birth records and were only included within ZIP-code level analyses. Missingness in covariates for the ZIP-code-level analysis was less than 1% of records (Supplementary Table S2). All protocols used in the study were approved by Virginia Tech Institutional Review Board (No. 16-898) and the Virginia Department of Health (No. 40221). 

### 2.2. Exposure Estimation

**Figure 1:**
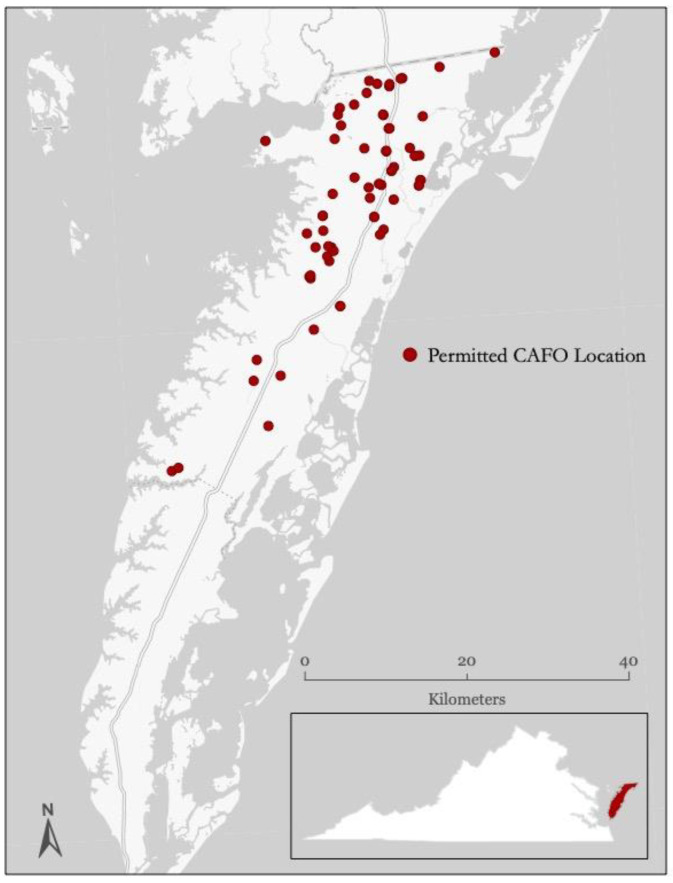
Locations of CAFOs on the Eastern Shore, Virginia (2011).

Information on poultry CAFO facilities was collected from the Department of Environment Quality of the Commonwealth of Virginia (Supplementary File S2). The dataset included the permit date of issuance, geocoded location of the CAFO, permit expiration date, and permit activity. Active poultry CAFOs ranged between 66 and 77, depending on the year, and were listed for every year that the permit was active, as shown in Figure 1. Individual CAFO operation locations were relatively stable through the study period (+/− 1–4 poultry CAFOs going offline, coming online in a given year). The prenatal exposure assignment for a birth was determined by the year in which the majority of gestation occurred (≥50% of gestation). For the ZIP-code-level analysis, the number of active poultry CAFOs contained within the maternal-address ZIP code during the majority gestation year for each birth was calculated. Births were categorized into non-exposed (no CAFO facilities within maternal ZIP code during the year of majority gestation) and exposed (1+ poultry CAFO facilities within maternal ZIP code during the year of majority gestation). 

For the street-level analysis, we constructed 3 buffer distances by using the *sf* package in R software, representing a radius of 1 km, 2 km, and 5 km surrounding each geocoded maternal address [34]. Previous spatial epidemiology studies have used buffers with varying distances to assess exposure to CAFOs, including 1 km, 2 km, 5 km, and up to 15 km [31,35,36]. Since the Eastern Shore of Virginia is a relatively small land area, buffer distances of 1 km, 2 km and 5 km were chosen. Births were considered “exposed” at each distance if a CAFO facility was contained within this diameter. Births occurring with maternal residential addresses containing no CAFO facilities within 5 km served as the reference (unexposed) population. An inverse distance weighted (IDW) approach was also used to estimate maternal exposure to account for distances from multiple CAFOs. This approach has been used to estimate individual air-pollutant exposure from multiple fixed locations [37]. Briefly, this method assumes that multiple locations of CAFOs within a buffer are not equally associated with birth outcomes, but instead those that are closer to the maternal residence will have a greater effect on birth outcomes, where the weighted count of active poultry facilities around each maternal address within the majority year of gestation was defined as follows:

$$\sum_{i=1}^{n}\frac{1}{d_{i}}$$

where n represents the number of existing poultry CAFOs surrounding maternal residence during the majority gestation year, and $d_{i}$ represents the distance of the $i_{\mathrm{th}}$ individual poultry CAFO from maternal residence. For example, an inversely distance weighted CAFO count of 6 CAFOs/km could be computed from (i) 6 CAFOs located within 1 km of maternal residence or (ii) 3 CAFOs located 0.5 km from the maternal residence. No buffer was used in this model. For subsequent statistical models, we classified IDW values into tertiles to explore exposure, defined as low (0–2.8 CAFOs/unit km circle), mid (2.8–6.24 CAFOs/unit km circle), or high exposure (6.24–13.8 CAFOs/unit km circle). 

### 2.3. Statistical Analysis 

Birth weight, gestational weeks, preterm birth (PTB), and low birth weight (LBW) were dependent variables in separate logistic and linear regression models, using the maternal street address or ZIP-code-level exposure estimates following the general equation below.

(1) $$\mathrm{Pr}\left(Y_{i}=1\right)=logit^{-1}\left(a+\beta_{p}P_{i}+\left(bs\left(t\right)\right)+\sum_{\kappa }\delta_{k}x_{ik}\right),\;i=1,\ldots ,n;k=1,\ldots ,K$$

where *Y* is the dichotomous birth outcome being modeled; $P_{i}$ is the number of CAFOs within the buffer around the maternal residence split into tertiles; $\beta_{p}$ is the beta coefficient of the birth outcome of interest; bs(t) represents the year (2002–2015), using a spline with 4 degrees of freedom; and $x_{ik}$ represents residual errors in the model (k covariates described below for individual i). To allow for secular, nonlinear trends in birth outcomes, we used the splines package in R software, incorporating inflection points (or splines) where fixed effects could vary [38]. Similar approaches have been utilized to characterize associations between birth outcomes and other environmental exposures [39,40,41]. 

Preterm birth was defined as less than a gestational age of 37 weeks, while low birth weight was defined as less than 2500 g total birth weight. Logistic regression models were used to predict odds of PTB/LBW, while linear regression models were used to examine the association between continuous birthweight/gestational weeks and maternal residence proximity to poultry CAFOs. Covariates included in the models included child sex; mother’s reported race, age, ethnicity, and education; self-reported tobacco use during pregnancy, method of payment for birth services, and previous births. Mother’s education was classified as not completing high school, high school completed, or college completed. Mother’s age was classified as those under 18, 18–35, and those above 35 years of age. Categories are based on prior studies showing an increased risk of adverse birth outcomes in the younger and older categories of maternal age [42]. Parity was determined from the child’s birth order. Parity was classified into one, two, three, or four or more births based on previous studies determining the relationship between parity and fetal outcomes [43,44]. Method of payment was included as an indicator of socioeconomic status and consisted of Medicaid, private insurance, self-paid, and other [45,46]. Maternal race reported on the birth record was grouped into White, Black, or Other as sample sizes were small for reported race other than Black and White. Ethnicity was classified as being Hispanic or not Hispanic based on the origin of the mother field in the birth record. All data processing and statistical analyses were performed in R [47], and packages sf and spline were used for spatial analysis and modeling splines, respectively [34,38].

## 3. Results

The study area and locations of poultry farms within the study area are illustrated in Figure 1. As shown in Table 1, demographics were similar across exposure groups, although the use of Medicaid was greater in the highest tertile of CAFO exposure, as compared to the lower tertiles, and private-insurance use was greater in the lowest tertile. Mother’s Hispanic classification was also greater in the highest tertile of exposure, as compared to the lowest tertile (Table 1). Similar trends were observed by using the buffer model (Supplementary Table S1). In comparing demographics from the ZIP-code-level analysis to the reduced set with street-level geocoded maternal addresses, minimal differences are evident, although the proportion of infants from mothers reporting as Hispanic is smaller in the street-level dataset (Supplementary Table S2).

In the ZIP-code-level analysis, a small non-significant increase in the odds of preterm birth and low birthweight, as well as a small decrease in birthweight and gestational weeks, was seen (Supplementary Table S3). Although not significant, a decrease in the direction expected was observed. Specifically, a decrease of 25.5 g (95% Confidence Interval: −55.9 g, 4.86 g) in birthweight and a decrease of 0.42 (95% CI: −1.33, 0.42) in gestational days were observed when at least one active poultry CAFOs was within the maternal-address ZIP code (Supplementary Table S3). Having an active poultry CAFO within the maternal address ZIP code was associated with 9% higher odds for low birthweight (Supplementary Table S3). 

In the street-level analysis, a decrease in birth weight was found when active poultry CAFOs were within 5 km of maternal residence (Supplementary Table S4). Specifically, maternal addresses with at least 1 CAFO between 2 and 5 km was associated with a decrease of 47.3 g (95% CI: −94.1, −1.70) in birthweight (Supplementary Table S4). No significant decrease in birthweight was found when maternal addresses had at least 1 CAFO between 0 and 1 km. 

The final model refined the exposure metric by using the inverse distance weighted approach, incorporating both the density and distance of CAFOs to the maternal address, and resulted in statistically significant associations for both birthweight (*p*-value = 0.01) and gestational days (*p*-value = 0.01), while the buffer model suggested a significant association for birthweight only (*p*-value = 0.04). A decrease in birthweight and gestational weeks was found when making comparisons in the low-to-high-exposure tertile (IDW= 2.8–6.2) (Table 2). Compared to births in the first exposure tertile, we found a decrease of 52.8 g (95% CI: −95.8, −9.8, *p* = 0.01) in birthweight and a decrease of 1.51 gestational days (95% CI: −2.78, −0.25, *p* = 0.01) (Table 2). The odds of preterm birth and LBW in the second and third tertiles of exposure were not statistically different from the first tertile (Table 2). The highest exposure tertile was associated with a non-significant 17% (95% CI: −6%, 44%) higher odds for preterm birth and 7% (95% CI: −14%, 34%) higher odds for low birth weight compared to the low tertile. Effect estimates of covariates in IDW models for birthweight and gestation length (Supplementary Tables S5 and S6) were consistent with findings from previous studies. For example, mothers who reported tobacco use during pregnancy had lower birthweights and shorter gestations, and mothers who identified as Black had infants with lower birthweights and shorter gestations [48,49,50]. 

## 4. Discussion

This study assessed birth outcomes on the Eastern Shore, Virginia, and suggests that maternal residency near active poultry feeding operations may be associated with reduced gestation and birth weight. Previous research has found that residential proximity to CAFOs is associated with poor health outcomes, particularly causing adverse respiratory symptoms [51,52,53,54]. This is the first study assessing poultry CAFOs and geospatial associations with birth outcomes in Virginia by using an IDW model. Similar results were found in a study in North Carolina assessing hog and poultry CAFOS and the geospatial associations with infant birth outcomes [31]. This study examined birth outcomes in the year 2016, while our study examined birth outcomes over a 13-year period (2002–2015). Although the study period was longer in this study, the NC study had a larger sample size due to the greater population size in the region studied. An inverse distance weighted model was used in our study to account for the distance and density of CAFOs from the maternal residence and offers a more precise measurement than buffer models, which have been used in other studies [35]. Our study, however, did not consider the number of animals or the amount of land area occupied by the CAFOs due to data unavailability. A small decrease in birth weight and gestational days was observed in both the present study and the study completed in North Carolina. Small decreases in average birthweight or gestational days at the population level can lead to increases in preterm births and low-birth-weight births. While the study in North Carolina found that maternal residency near CAFOs was associated with an increased odds of preterm birth and low birth weight, the present study detected a small but non-significant increase in the odds of preterm birth and low birth weight, and no differences were detected when examining different levels of proximity to CAFOs. 

While future studies should examine birth outcomes in other areas near poultry/other agricultural animal-feeding operations, such as in Maryland and in the Midwest, prior research has shown that CAFOs are located in areas with higher proportions of persons identifying as Black or Hispanic and in low-income areas [55,56,57]. A study in Mississippi found that there were 2.4–3.6-times more CAFOs next to census block groups with high percentages of residents identifying as African American and low-income [58]. In the present study, mothers identifying as Hispanic and birth services supported by Medicaid are greatest in the highest tertile of exposure in this study (Table 1). Hence, exposure disparities may be contributing to socioeconomic and race/ethnicity disparities in health. 

Limitations of the current analysis include potential confounding by variables that were not available in the dataset, or residual confounding due to imperfect covariate or exposure measurement. For example, we did not have information on the mother’s prenatal care, nutrition, disease status, nontobacco drug exposures, body mass index, proximity to other sources of pollutants, and employment status. As shown in Figure 1, many of the poultry CAFOs are concentrated in the Northern part of the Eastern Shore (Accomack County). The regional hospital located on the Eastern Shore is in Accomack County. Therefore, mothers who reside in the Southern part of the Eastern Shore (Northampton County) may experience decreased access to healthcare, and this may be a contributing factor to decreased birth weights observed at the 2–5 km buffer and not in the 1 km buffer of the street-level analysis. Secondly, we were unable to track maternal mobility during the gestational period. We assumed that the mother stayed in the same residence she reported on the birth records during the whole gestation period, which could result in exposure misclassification. Studies have shown that most mothers stay within the same region during pregnancy, and if they move, it is within the same locality [59,60]. Although occupational mobility could also lead to exposure misclassification, studies have shown that occupational mobility has a small impact on environmental exposure levels [61].

Our study used an inverse distance weighted model, which examined the density and distance of CAFOs. Although this method allows for a more refined exposure metric than methods used in previous studies, it does not fully assess CAFO exposure. Air dispersion models, such as the Industrial Source Complex Short-Term Model, (ISC-ST3) and the California Puff Model (CALPUFF) model, have been used by other studies to model ammonia and hydrogen sulfide emissions from CAFOs and account for wind-direction and land-cover pattern [62,63]. Further research is needed, however, focusing on modeling poultry CAFO exposure, as these models assessed hog and swine CAFO exposure. Furthermore, improper waste management practices of CAFOs lead to decreased water quality [64]. Microbial pathogens, pharmaceuticals, and excessive nutrients present in the waste contaminate water resources. High levels of nitrates found in water have been linked to an increased risk of methemoglobinemia, otherwise known as “blue baby syndrome”. Thus, a more refined exposure metric can be used that considers potential sources of contamination of well- and municipal-water sources. There are many pathways through which CAFOs could influence health, thus making the estimation of CAFO exposure and modeling of agricultural air quality complex. Future research should aim to assess CAFO exposure by using a more advanced method that considers wind direction, speed, land cover, water sources, and other geographic and meteorological parameters. 

## 5. Conclusions

Our findings suggest that there is an association between maternal residency near active poultry feeding operations and reduced gestation and birth weight. We further found that the inverse distance weighted model, which weights increasing numbers of CAFOs closer to the maternal residence, is more strongly associated with adverse birth outcomes. This study adds to the growing body of works in the literature exploring proximity to CAFOs and adverse birth outcomes. 

## Figures and Tables

**Table 1:** Demographics of mother and child by low, medium, and high categories of inverse distance weighted (IDW) proximity to CAFOs *. The unit of IDW is the number of CAFOs per unit km circle.

	Low (IDW = 0–2.8) (N = 1902)	Medium (IDW = 2.8–6.24) (N = 1903)	High (IDW = 6.24–13.8) (N = 1963)
Characteristic			
Child’s sex	N (%)	N (%)	N (%)
Male	971 (51.1)	993 (52.2)	1021(52.0)
Female	931 (48.9)	910 (47.8)	942 (48.0)
Mother’s race			
White	997 (52.4)	1190 (62.5)	1214 (61.8)
Black	870 (45.7)	661 (34.7)	687 (34.9)
Other	34 (1.79)	50 (2.63)	53 (2.70)
NA	1 *	2 *	9 *
Mother’s age			
18–35	1630 (85.7)	1591 (83.6)	1688 (86.0)
<18	132 (6.94)	149 (7.83)	159 (8.10)
>35	140 (7.36)	163 (8.57)	116 (5.90)
Previous births			
1	753 (39.5)	706 (37.1)	691 (35.2)
2	642 (33.8)	606 (31.8)	620 (31.6)
3	310 (16.3)	338 (17.8)	381 (19.4)
4	197 (10.4)	253 (13.3)	271 (13.8)
Mother’s education			
High school not completed	523 (27.5)	764 (40.1)	900 (45.8)
High school completed	688 (36.2)	599 (31.5)	669 (34.1)
College completed	691 (36.3)	540 (28.4)	394 (20.1)
Reported tobacco use during pregnancy			
No	1670 (87.8)	1665 (87.5)	1796 (91.5)
Yes	79 (4.15)	65 (3.42)	57 (2.90)
NA	153 (8.04)	173 (9.09)	110 (5.60)
Payment			
Medicaid	1139 (59.9)	1189 (62.5)	1341 (68.3)
Private insurance	626 (32.9)	522 (27.4)	388 (19.8)
Self-pay	134 (7.05)	191 (10.0)	230 (11.7)
NA	3 *	1 *	4 *
Mother’s Hispanic origin			
Hispanic	249 (13.1)	477 (25.1)	574 (29.2)
Non-Hispanic	1650 (86.7)	1425 (74.9)	1382 (70.4)
NA	3 *	1 *	7 *

* Less than 1%.

**Table 2:** Associations between birth outcomes and proximity to poultry CAFOs quantified by using IDW model. In parentheses, 95% confidence intervals are provided in.

Outcome Variable	Active Poultry CAFO (Second Tertile (2.8–6.24) *)	Active Poultry CAFO (Third Tertile (6.24–13.8) *)
Birth weight (g)	−15.7 (−58.7, 27.3) ^a^	**−52.8 (−95.8, −9.8) ^a^**
Gestational days	−0.73 (−1.99, 0.52) ^b^	**−1.51 (−2.78, −0.25) ^b^**
Preterm	1.01 (0.81, 0.1.25) ^c^	1.17 (0.94, 1.44) ^c^
Low birth weight	0.95 (0.76, 1.18) ^c^	1.07 (0.86, 1.34) ^c^

* Unit: number of CAFOs per unit km circle. ^a^ Change in birth weight (g). ^b^ Change in gestational days. ^c^ Odds ratio.

## Data Availability

Data-use agreements with state health agencies prohibit authors from sharing birth-record data used in the analysis. Poultry-farm data were acquired from the Virginia Department of Environmental Management and are included in Supplementary File S2.

## Supplementary Materials

The following supporting information can be downloaded at https://www.mdpi.com/article/10.3390/healthcare10102016/s1. Table S1: Demographics buffer model. Table S2: Demographics of street-level and ZIP-code analysis. Table S3: Associations between birth outcome and poultry CAFOs within maternal-residence ZIP code. Table S4: Demographics of street-level and ZIP-code analysis. Association between birth outcomes and exposure to poultry CAFOs: buffer model. Table S5: Effect estimates of covariates included in IDW model: birthweight (g). Table S6: Effect estimates of covariates included in IDW model: gestation length (days).
