# Supplementary material for: Poultry Concentrated Animal-Feeding Operations on the Eastern Shore, Virginia, and Geospatial Associations with Adverse Birth Outcomes
Source: Healthcare (Basel). 2022 Oct 12;10(10):2016. doi: 10.3390/healthcare10102016 (PMC9602095; doi:10.3390/healthcare10102016)
Supplement: Supplementary file 1 [file healthcare-10-02016-s001.zip › healthcare-1901531-supplementary/Supplementary Tables.pdf]

Supplementary materials:

Supplemental Table S1.
Demographics: Buffer Model

Characteristic	Active Poultry within 1 km (N=1,966)	Active Poultry within 1-2 km (N=1,080)	Active poultry within 2-5 km (N=1,341)	No active poultry within 5km (N=1,381)
Child's sex	**N (%)**	**N (%)**	**N (%)**	**N (%)**
Male	1,018 (51.8)	572 (53.0)	690 (51.5)	705 (51.0)
Female	948 (48.2)	508 (47.0)	651 (48.5)	676 (49.0)
Mother's race				
White	1,118 (56.9)	704 (65.2)	835 (62.3)	744 (53.9)
Black	812 (41.3)	341 (31.6)	468 (34.9)	597 (43.2)
Other	35 (1.8)	30 (2.8)	34 (2.5)	38 (2.8)
NA	1	5	4	2
Mother's age				
18-35	1,698 (86.4)	934 (86.5)	1,140 (85.0)	1,137 (82.3)
<18	132 (6.7)	82 (7.6)	108 (8.1)	118 (8.5)
>35	136 (6.9)	64 (6.9)	93 (6.9)	126 (9.1)
Previous births				
1	782 (39.7)	396 (36.7)	469 (35.0)	503 (36.4)
2	659 (33.5)	336 (31.1)	431 (32.1)	442 (32.0)
3	324 (16.5)	202 (18.7)	247 (18.4)	256 (18.5)
4	201 (10.2)	146 (13.5)	194 (14.5)	180 (13.0)
Mother's education				
Did not complete high school	544 (27.7)	517 (47.9)	642 (47.9)	484 (35.0)
High school completed	738 (37.5)	342 (31.7)	400 (29.8)	476 (34.5)
College completed	684 (34.8)	221 (20.5)	299 (22.3)	421 (30.5)
Reported tobacco use during pregnancy				
No	1731 (88.0)	976 (90.4)	1,221 (91.1)	1203 (87.1)
Yes	85 (4.3)	27 (2.5)	35 (2.8)	54 (3.9)
NA	150 (7.6)	77 (7.1)	85 (6.3)	124 (9.0)
Payment				
Medicaid	1,195 (60.8)	740 (68.5)	884 (65.9)	850 (61.5)
Private insurance	631 (32.1)	205 (19.0)	293 (21.8)	407 (29.5)
Self-pay	138 (7.0)	134 (12.4)	162 (12.1)	121 (8.8)
NA	2	1	2	3
Mother's Hispanic origin				
Not Hispanic	1,723 (87.6)	714 (66.1)	903 (67.3)	1,117 (80.9)
Hispanic	241 (12.3)	364 (33.7)	436 (32.5)	259 (18.8)
NA	2	2	2	5

NA indicates not available

Supplemental Table S2.
Demographics of street-level and zip-code analysis

Characteristic	Street-level N=5,768	Zip-code N=7,306
Child's sex		
Male	2,985 (51.8)	3,803 (52)
Female	2,783 (48.2)	3,503 (48)
Mother's race		
White	3,401 (59.0)	4,366 (59.8)
Black	2,218 (38.5)	2,747 (37.6)
Other	137 (2.4)	175 (2.4)
NA	12	18
Mother's age		
18-35	4,909 (85.1)	6,199 (85)
<18	440 (7.6)	571 (7.8)
>35	419 (7.3)	536 (7.3)
Previous births		
1	2,150 (37.3)	2,694 (36.9)
2	1,868 (32.4)	2,333 (31.9)
3	1,029 (17.8)	1,207 (16.5)
4	721 (12.5)	972 (13.3)
Mother's education		
Did not complete high school	2,187 (37.9)	2,644 (36.2)
High school completed	1,956 (33.9)	2,575 (35.2)
College completed	1,625 (28.2)	2,060 (28.2)
Reported tobacco use during pregnancy		
No	5131(89.0)	6551(89.6)
Yes	201 (3.5)	230 (3.4)
NA	436	525
Payment		
Medicaid	3,668 (63.6)	4,724 (64.7)
Private insurance	1,536 (26.7)	1,851 (25.3)
Self-pay	555 (9.6)	720 (9.9)
NA	8	11
Mother's Hispanic origin		
Not Hispanic	4, 557 (79.0)	5,399 (74)
Hispanic	1,200 (20.8)	1,893 (25.9)
NA	11	14

NA indicates not available

Zip-Code Analysis:

Supplemental Table S3.
Associations between birth outcome and poultry CAFOs within maternal residence zip code. 95% confidence intervals are shown.

Outcome variable	Association with active poultry CAFOs within maternal address zip code
Birthweight	-25.5 (-55.9, 4.86)[a]
Gestational days	-0.42 (-1.33, 0.42)[b]
Preterm	1.00 (0.86, 1.20)[c]
Low birth weight	1.09 (0.92, 1.30)[c]

[a]Change in birth weight (grams); [b] Change in gestational days; [c]Odds ratio

Street-level buffer Analysis:

Supplementary Table S4.
Association between birth outcomes and exposure to poultry CAFOs: Buffer Model

Outcome variable	Active poultry CAFO within 1 km	Active poultry CAFO between 1-2 km	Active poultry CAFO between 2-5 km
Birthweight	-47.4 (-97.5, 3.62)[a]	-19.7 (-69.6, 24.6)[a]	**-47.3 (-94.1, -1.70)[a]**
Gestational days	-1.00 (-2.52, 0.49)[b]	-0.49 (-1.89, 0.91)[b]	-1.25 (-2.59, 0.10)[b]
Preterm	1.05 (0.80, 1.37)[c]	1.53 (0.91, 1.46)[c]	1.20 (0.95, 1.51)[c]
Low birth weight	1.08 (0.83, 1.42)[c]	1.12 (0.88, 1.44)[c]	1.19 (0.80, 1.29)[c]

[a]Change in birth weight (grams); [b] Change in gestational days; [c]Odds ratio

Supplemental Table S5.
Effect estimates of covariates included in IDW model: birthweight (grams)

Covariate	Change in birthweight (grams)	P-value	95% CI
**Age of mother**			
<18	-48.3	.177	-118,21.7
>35	2.28	.949	-67.5,72.0
**Race**			
Black	-355	5.20E-64	-396,-315
Other	-227	1.06E-4	-343,-113
**Method of payment**			
Private	11.9	.606	-33.3,57.1
Self-pay	-68.6	.025	-128,-8.78
**Mother's Hispanic origin**			
Yes	-9.37	.733	-63.4,44.6
**Reported tobacco use during pregnancy**			
Yes	-237	1.31E-06	-334,-141
**Mother's education**			
High school completed	84.4	3.56E-4	38.1,131
College completed	71.4	8.56E-3	18.2,125
**Sex of child**			
Female	-129	2.49E-13	-163,-94.5
**Previous births**			
2	89.3	3.95E-05	46.8,132
3	82.0	1.84E-3	30.4,134
4	33.8	.276	-27.0,94.6

Supplemental Table 6.

Effect estimates of covariates included in IDW model: gestation length (days)

Covariate	Change in gestation (days)	P-value	95% CI
**Age of mother**			
<18	-1.42	.175	-.498,.090
>35	-.167	.873	-.317,.269
**Race**			
Black	-5.79	3.10E-21	-.997,-.656
Other	-7.00	4.83E-05	-1.48,-.519
**Method of payment**			
Private	-3.12	4.00E-06	-.636,-.257
Self-pay	-3.92	1.27E-05	-.812,-.309
**Mother's Hispanic origin**			
Yes	.967	.234	-.089,.366
**Reported tobacco use during pregnancy**			
Yes	-4.15	.004	-.966,-.189
**Mother's education**			
High school completed	.684	.325	-.097,.292
College completed	-.267	.738	-.261,.185
**Sex of child**			
Female	.132	.799	-.126,.163
**Previous births**			
2	-1.37	.031	-.375,-.018
3	-2.55	9.68E-4	-.581,-.148
4	-3.99	1.20E-05	-.825,-.315
